# Zhishi Daozhi decoction alleviates constipation induced by a high-fat and high-protein diet via regulating intestinal mucosal microbiota and oxidative stress

**DOI:** 10.3389/fmicb.2023.1214577

**Published:** 2023-09-18

**Authors:** Xinxin Peng, Xin Yi, Na Deng, Jing Liu, Zhoujin Tan, Ying Cai

**Affiliations:** ^1^The First Affiliated Hospital of Hunan University of Chinese Medicine, Changsha, China; ^2^The Domestic First-Class Discipline Construction Project of Chinese Medicine, Hunan University of Chinese Medicine, Changsha, China

**Keywords:** Zhishi Daozhi decoction, high-fat and high-protein diet, microbial diversity, intestinal mucosal microbiota, constipation

## Abstract

**Background:**

A growing body of evidence has demonstrated that a high-fat and high-protein diet (HFHPD) causes constipation. This study focuses on understanding how the use of Zhishi Daozhi decoction (ZDD) affects the intricate balance of intestinal microorganisms. The insights gained from this investigation hold the potential to offer practical clinical approaches to mitigate the constipation-related issues associated with HFHPD.

**Materials and methods:**

Mice were randomly divided into five groups: the normal (MN) group, the natural recovery (MR) group, the low-dose ZDD (MLD) group, the medium-dose ZDD (MMD) group, and the high-dose ZDD (MHD) group. After the constipation model was established by HFHPD combined with loperamide hydrochloride (LOP), different doses of ZDD were used for intervention. Subsequently, the contents of cholecystokinin (CCK) and calcitonin gene-related peptide (CGRP) in serum, superoxide dismutase (SOD), and malondialdehyde (MDA) in the liver were determined. The DNA of intestinal mucosa was extracted, and 16S rRNA amplicon sequencing was used to analyze the changes in intestinal mucosal microbiota.

**Results:**

After ZDD treatment, CCK content in MR group decreased and CGRP content increased, but the changes were not significant. In addition, the SOD content in MR group was significantly lower than in MLD, MMD, and MHD groups, and the MDA content in MR group was significantly higher than in MN, MLD, and MHD groups. Constipation modeling and the intervention of ZDD changed the structure of the intestinal mucosal microbiota. In the constipation induced by HFHPD, the relative abundance of pathogenic bacteria such as *Aerococcus, Staphylococcus, Corynebacterium, Desulfovibrio, Clostridium*, and *Prevotella* increased. After the intervention of ZDD, the relative abundance of these pathogenic bacteria decreased, and the relative abundance of *Candidatus Arthromitus* and the abundance of Tropane, piperidine, and pyridine alkaloid biosynthesis pathways increased in MHD group.

**Conclusion:**

Constipation induced by HFHPD can increase pathogenic bacteria in the intestinal mucosa, while ZDD can effectively relieve constipation, reduce the relative abundance of pathogenic bacteria, and alleviate oxidative stress injury. In addition, high-dose ZDD can increase the abundance of beneficial bacteria, which is more conducive to the treatment of constipation.

## 1. Introduction

Constipation is a common and onerous gastrointestinal disease (Dimidi et al., 2017). Its definition includes infrequent defecation, excessive tension, a feeling of incomplete defecation, failure, or excessive time spent on defecation attempts (Sharma and Rao, 2017). Eating habits are one of the important factors that cause constipation. Studies have shown that unhealthy diets, especially HFHPD and low-fiber diets, are important predisposing factors for constipation (Stewart and Schroeder, 2013; Tabbers et al., 2014). In contrast, the intake of dietary fiber and the appropriate amount of water can relieve the symptoms of constipation (Yang et al., 2012). Studies have found that a high-fat diet can cause indigestion, cause constipation, and delay colon transit time by reducing colon mucus in mice (Mukai et al., 2020). Moreover, Liu et al. (2022) found that under a high-fat diet, LOP-induced constipation in mice developed symptoms such as oxidative stress, gastrointestinal hypomotility, and intestinal neurotransmitter disorder. In a study involving older adults and children, high saturated fat intake was closely related to constipation (Lee et al., 2012; Taba Taba Vakili et al., 2015).

Numerous recent studies have shown that constipation is closely related to the intestinal microbiota. The feces in the intestine are stimulated by harmful substances and enrich the harmful bacteria (Zhang X. et al., 2021). Changes in the intestinal microbiota of patients with constipation are generally manifested by a relative decrease in beneficial bacteria and a potential increase in harmful bacteria. These changes may affect intestinal motility and intestinal secretion function by changing the amount of available physiologically active substances in the intestine and the intestinal metabolic environment (Staller et al., 2022). In addition, diet will also change the genetic composition and metabolic activities of the microorganisms living in our bodies. It can be deduced that a high-fat diet has an important relationship with the occurrence of chronic diseases such as constipation (Tan et al., 2021). LOP is a commonly used antidiarrheal in clinics to increase the consistency and hardness of stool to control the symptoms of acute and chronic diarrhea. Thus, LOP is suitable to serve as an inducer combined with HFHPD to establish a constipation animal model (Zhang et al., 2022).

ZDD is a prescription in traditional Chinese medicines derived from Li Dongyuan's Nei Wai Shang Bian Huo Lun and is used for the treatment of constipation and indigestion (Liu F. et al., 2020). Modern pharmacological research shows that ZDD can promote gastric emptying and small intestine propulsion to restore the function of the spleen and stomach transport (Li et al., 2008). This prescription consists of Aurantii Fructus Immaturus (*Citrus aurantium* L.), Rhei Radix et Rhizoma (*Rheum officinale Baill*.), Poria [*Poria cocos* (Schw.) Wolf], Scutellariae Radix (*Scutellaria baicalensis* Georgi), Coptidis Rhizoma (*Coptis chinensis* Franch.), Atractylodis Macrocephalae Rhizoma (*Atractylodes macrocephala* Koidz.), Alismatis Rhizoma [*Alisma orientate* (Sam.) Juzep.], and Massa Medicata Fermentata (Medicated Leaven) (Lin et al., 2020). Among them, Atractylodis Macrocephalae Rhizoma and Poria can improve gastrointestinal hormone secretion and promote gastric emptying. Alismatis Rhizoma has a hypoglycemic effect. Rhei Radix, and Rhizoma can inhibit intestinal water absorption, enhance intestinal peristalsis, and promote defecation. Aurantii Fructus Immaturus can recover intestinal contraction rhythm, improve gastrointestinal excitability, enhance gastrointestinal peristalsis, and relieve gastrointestinal spasms. Massa Medicata Fermentata can increase appetite and maintain the normal digestive function of the body. The combination of these drugs has a definite effect on gastrointestinal infectious inflammation with dyspepsia (Wang et al., 2018; Shen and Tan, 2022). The curative effect of decoction is more obvious than that of pills, and it takes effect faster (Zhu et al., 2022).

However, the relationship between the changes in intestinal mucosal microbiota induced by HFHPD and constipation remains unclear. Therefore, in this study, the constipation model of mice was induced by LOP under the condition of HFHPD. To study the changes in intestinal mucosal microbiota and the levels of gastrointestinal hormones and oxidative stress in mice with HPHFD-induced constipation under the intervention of ZDD.

## 2. Materials and methods

### 2.1. Materials

#### 2.1.1. Animals

To exclude the influence of gender on the study, we selected 50 specific-pathogen-free male mice as the research objects (Wu et al., 2022). Mice were purchased from Hunan Slaccas Jingda Laboratory Animal Co., Ltd.

#### 2.1.2. Feed

Self-made high-fat and high-protein feed consists of milk powder (Nestle family nutritious milk powder), flour (Huiyi gluten wheat flour), floss powder (Zhenqiao golden floss), and bean powder (Yonghe sweet soybean milk powder) in a ratio of 1: 1: 1: 2 (He et al., 2019).

The main indexes of nutritional components of growth feed include water, crude protein, crude fiber, crude fat, crude ash, calcium, total phosphorus, lysine, methionine, and cystine. It was provided by the Animal Experiment Center of the Hunan University of Chinese Medicine.

#### 2.1.3. Drugs

ZDD consists of 10 g of Aurantii Fructus Immaturus, 20 g of Rhei Radix et Rhizoma, 6 g of Coptidis Rhizoma, 6 g of Scutellariae Radix, 10 g of Massa Medicata Fermentata, 10 g of Atractylodis Macrocephalae Rhizoma, 6 g of Poria, and 4 g of Alismatis Rhizoma. All these ingredients were purchased from the First Affiliated Hospital of Hunan University of Chinese Medicine. The procedure involved soaking the aforementioned drugs in boiling water for 10 cycles, with each cycle lasting 10 min. The resulting mixture was then filtered and concentrated using a rotary evaporator at 75°C. This process yielded water-based decoctions with varying crude drug concentrations of 0.2, 0.4, and 0.8 g/mL.

LOP capsules was produced by Xian Janssen Pharmaceutical Co., Ltd. (Batch production No. MDJ7007).

### 2.2. Methods

#### 2.2.1. Grouping of experimental animals

All the mice were fed adaptively at a temperature of 23–25°C and a relative humidity of 50–70% in a clean and quiet environment for 3 days before modeling. The 50 mice were randomly divided into five groups: the normal (MN) group, the natural recovery (MR) group, the low-dose ZDD (MLD) group, the medium-dose ZDD (MMD) group, and the high-dose ZDD (MHD) group. All experiments and procedures involving animals were conducted in accordance with the protocol approved by the Institutional Animal Care and Use Committee of the Hunan University of Chinese Medicine.

#### 2.2.2. Construction of the constipation model

The procedure was divided into two stages. In the first stage, the model mice were fed with HFHPD and gavaged with 50 mg/kg milk twice a day. MN group was fed growth feed and gavaged with an equal dose of sterile water for 7 days (Mai et al., 2018). In the second stage, based on the first stage, the model mice were intraperitoneally injected with 3 mg/kg LOP for 7 days, once a day. MN group ate normally and was intraperitoneally injected with equal doses of normal saline for 7 days (Hajji et al., 2020).

#### 2.2.3. Intervention of ZDD

After the successful model, the model mice were divided into MR, MLD, MMD, and MHD groups. The mice in MLD, MMD, and MHD groups were gavaged with 2.4, 4.7, and 9.4 mg/kg ZDD, respectively, twice a day. MN and MR groups were gavaged with an equal dose of sterile water for 7 days.

#### 2.2.4. General characteristics

At the same time in the morning, the mice were observed and recorded in terms of fur, mental status, activity, fecal traits and odor, perianal cleanliness, and body weight (Li C. R. et al., 2022).

#### 2.2.5. Measurement of CCK and CGRP in serum and MDA and SOD in liver

We selected five mice with consistent fecal characteristics in each group for analysis, and whole blood samples were collected by eyeball extraction. The blood sample was allowed to stand at room temperature for 2 h before being centrifuged at low temperature and high speed (4°C, 3,000 r/min) for 10 min. The supernatant was used for CCK and CGRP content determination. The CCK and CGRP contents of serum were determined by enzyme-linked immunosorbent assay (ELISA). After the mice were killed by cervical dislocation on the aseptic operating platform, the liver was removed, and 0.1 g of liver tissue was collected. Then, 1 mL of normal saline was added to the liver tissue in the ice bath for homogenization, and the mixture was centrifuged at 8,000 g, 4°C for 10 min. The MDA and SOD contents in liver homogenate were determined by ELISA, and the specific operation was carried out according to the instructions of the kit. The MDA kit was provided by Beijing Solarbio Science & Technology Co., Ltd. The CCK, CGRP, and SOD kits were provided by Quanzhou Kenuodi Biotechnology Co., Ltd.

#### 2.2.6. Extraction of intestinal mucosal samples

In each group, five mice were chosen based on consistent fecal characteristics. These mice were euthanized through cervical dislocation on a sterile operating table, and segments spanning from the jejunum to the ileum were collected. After the contents of the bowel segment were extruded, the bowel was cut longitudinally. Then, the residual contents in the intestinal wall were washed with normal saline, and the normal saline on the intestinal wall was sucked dry with filter paper. The intestinal mucosa was scraped off with a cover glass and placed in an EP tube at −80°C for subsequent storage (Zhang et al., 2020).

#### 2.2.7. PCR amplification and illumina Novaseq metagenome sequencing

PCR amplification was conducted using bacteria-specific primers targeting the 16S rRNA V3+V4 region. The primer sequences used were as follows: Forward primer 338F (5′-barcode + ACTCCTACGGGAGGCAGCA-3′) and reverse primer 806R (5′-GGACTACHVGGGTWTCTAAT-3′). The template DNA was pre-denatured at 98°C for 30 s using the PCR instrument, ensuring its completed denaturation for the subsequent amplification cycle. Within each cycle, the template was subjected to a 15-s denaturation step at 98°C, followed by a 30-s primer annealing step at 50°C to ensure optimal primer-template binding. The temperature was then maintained at 72°C for 30 s, facilitating primer extension, DNA synthesis, and completion of a single cycle. This cycle was iterated between 25 an 27 times to accumulate a substantial quantity of amplified DNA fragments. Finally, the product was kept at 72°C for 5 min so that it was completely extended and preserved at 4°C. The amplification results were subjected to 2% agarose gel electrophoresis, and the target fragments were cut out, and then, the target fragments were recovered using the Axygen gel recovery kit. Then, 2 × 250 bp double-ended sequencing was performed on the Illumina NovaSeq machine using the Novaseq 6000 SP Reagent Kit (500 cycles). Sample DNA extraction, amplification, and library sequencing were completed by Shanghai Personalbio Technology Co., Ltd.

#### 2.2.8. Bioinformatics

The 16S rRNA high-throughput sequencing was used to analyze the intestinal mucosal microbiota, and the modified and improved process was used to analyze the biological information of the microbiota. Moreover, 100% sequence similarity was merged to generate characteristic sequence amplicon sequence variants (ASV). The ASV table was used to draw a species accumulation curve, which is used to detect the sequencing depth and evaluate the quality of the sequence data (Qiao et al., 2023). At the same time, various metrics at the ASV level, including Chao 1, observed categories, Shannon, and Simpson indices, were calculated and analyzed based on the ASV table. Beta diversity analysis uses the Bray–Curtis distance to analyze the structural changes of microbial communities between samples and uses non-metric multidimensional scaling (NMDS) for visualization (Li X. Y. et al., 2022). The linear discriminant analysis (LDA) effect size (LefSe) method is used to detect classification units with rich differences between groups. To evaluate the diagnostic efficiency of different genera selected by LefSe analysis, the statistically significant receiver operating characteristic (ROC) curves of different genera were constructed, and the area under the curve (AUC) was calculated. We calculated the Spearman correlation coefficient, constructed a correlation heat map, and explored the relationship between different microbiota. The metabolic function of microbial microbiota was predicted using PICRUSt2 on the Kyoto Encyclopedia of Genes and Genomes (KEGG) (https://www.kegg.jp/) database (Douglas et al., 2020).

#### 2.2.9. Statistical analysis

SPSS 25.00 software was used for statistical analysis, and the data of each group were expressed as the mean ± standard deviation. GraphPad Prism 9 was used to draw histograms and box charts. If the two groups of data conform to a normal distribution and homogeneity of variance, the ANOVA test is used. The non-parametric test was used if it did not meet the requirements. A *P-*value of <0.05 was statistically significant.

## 3. Results

### 3.1. Effects of different doses of ZDD on the general characteristics of mice with HPHFD-induced constipation

**Figure 1:**
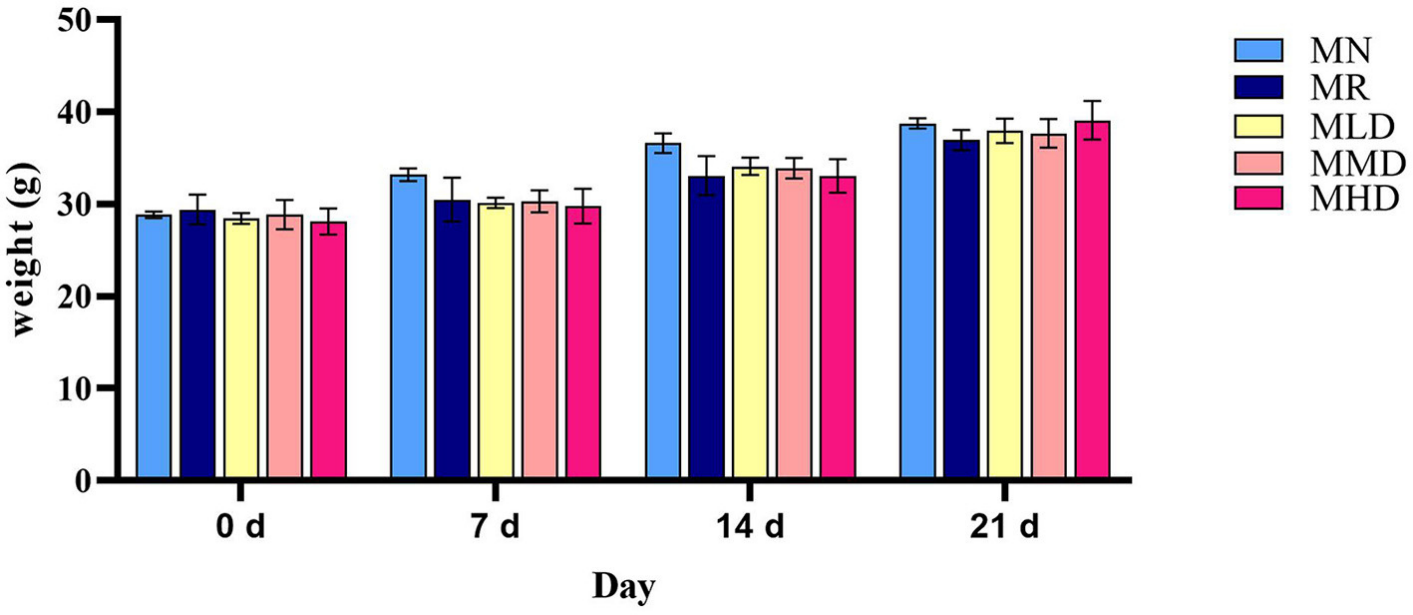
Changes of body weight in mice.

Before modeling, the mice in each group had glossy fur and good autonomous activity. After the modeling was completed, the model mice had withered fur and liked to gather, and their autonomous activity ability was reduced. After the intervention of ZDD, the fur was smooth, and the ability to make independent movements was improved. However, the fur of MR group was yellow, and the mental activity was poor. As can be observed from Figure 1, before modeling (0 d), the weight of mice in each group was similar, and in the first (7 d) and second modeling stages (14 d), the weight of model mice was lower than MN group. However, after the administration of ZDD (21 d), the weights of the mice in the MLD, MMD, and MHD groups increased more than that of the mice in the MR group, though not significantly, and the weight of the mice in the MHD group was closer to that of the mice in the MN group.

Before modeling, the feces of mice in each group were similar in appearance, shaped with moderate hardness, and turned dark brown. After the first stage of modeling, the feces of model mice were shaped but soft, sticky, and yellow. After the second modeling stage, the model mice had feces characterized by a beaded appearance. These feces were sticky and soft and exhibited signs of being incomplete during defecation, requiring more time for the process. After the administration of ZDD, the feces of MLD, MMD, and MHD groups had a moderate level of hardness, which was similar to that of the MN group. However, the feces of the mice in the MR group were harder notably harder than those of the MN group, with a rough, uneven, and dark brown appearance. Given the characteristics of incomplete defecation and the extended duration of the second modeling stage, we believe that the modeling process was successful.

### 3.2. Effects of different doses of ZDD on the contents of CCK and CGRP in the serum of mice with HPHFD-induced constipation

**Figure 2:**
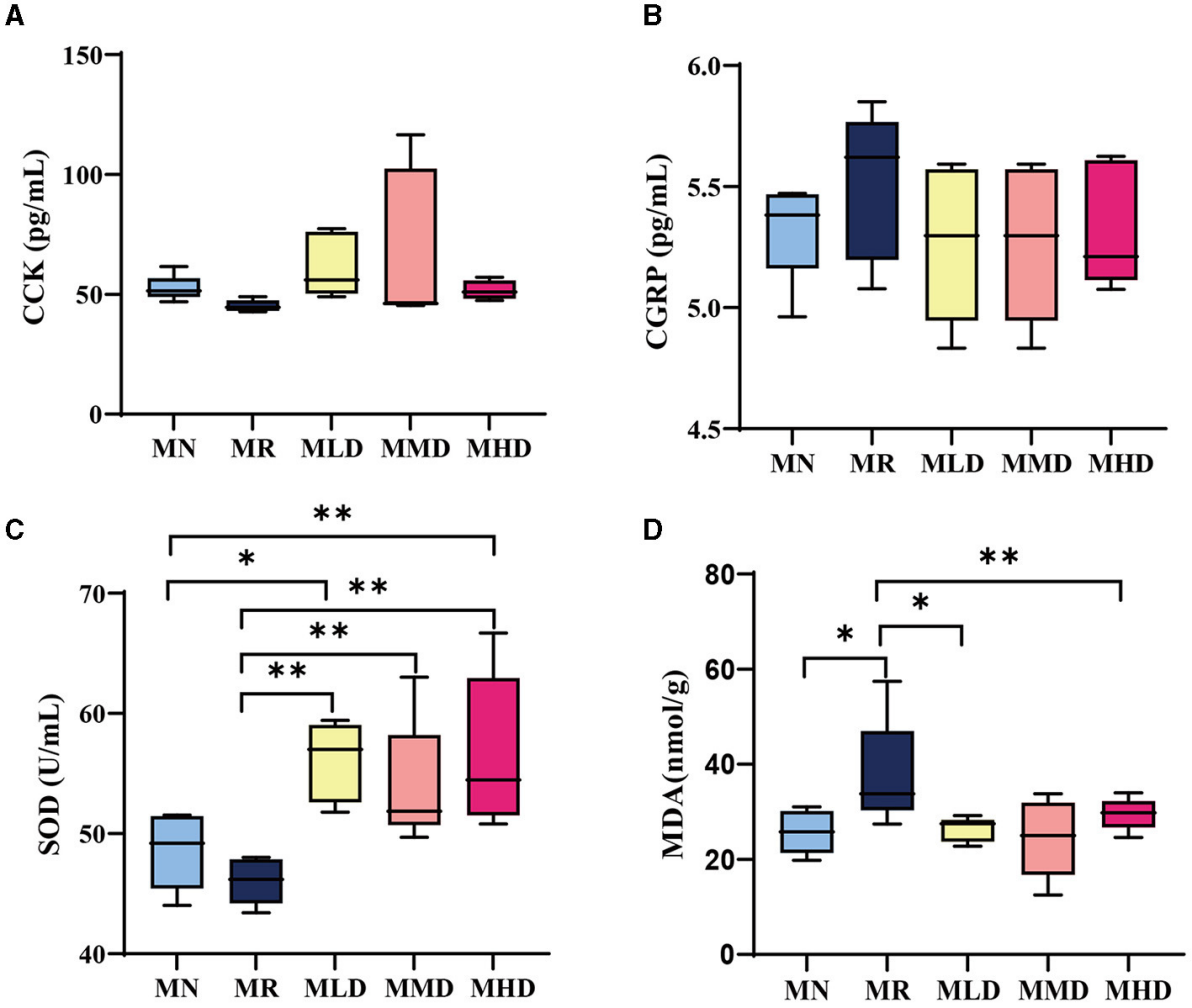
Gastrointestinal hormone and oxidative stress level of mice. **(A)** CCK content in serum. **(B)** CGRP content in serum. **(C)** SOD content in the liver. **(D)** MDA content in the liver. *n* = 5, *p < 0.05, **p < 0.01.

CCK is secreted by a large number of cells in the central nervous system, duodenum, and jejunum mucosa of mammals. It stimulates the contraction of the gallbladder and relaxation of the sphincter of Oddi to regulate the movement of the small intestine and colon (Wang et al., 2021). Figure 2A shows the CCK content of mice. Though not significantly, the CCK content in MR group was lower than that in MLD, MMD, and MHD groups. In particular, the CCK content in MHD and MN groups was similar. It could be speculated that ZDD had a certain recovery effect on CCK content in constipated mice.

CGRP, mainly distributed in the visceral sensory nerves, inhibits most gastrointestinal movement and is an important neurotransmitter for regulating the function of the digestive tract (Zhang et al., 2018). As shown in Figure 2B, compared to the MR group, the CGRP content in the MLD, MMD, and MHD groups decreased but not significantly. It can be shown that the intestinal peristalsis ability of mice had been restored to some extent after the administration of ZDD.

### 3.3. Effects of different doses of ZDD on the contents of SOD and MDA in the liver of mice with HPHFD-induced constipation

Oxidative stress has a strong cytotoxic effect on the body, which can damage the intestinal mucosal cells and cause intestinal mucosal dysfunction or induce or aggravate constipation (Xiang et al., 2019; Yi et al., 2023). SOD is an important antioxidant enzyme in the body and can play an antioxidant role (Kim et al., 2021). Figure 2C shows that the SOD content in the mice of the MR groups is significantly lower than that of the MLD, MMD, and MHD groups. At the same time, the SOD content in the MN group is significantly lower than that in the MLD and MHD groups. MDA is one of the products of lipid peroxidation, and its concentration can reflect the degree of cell damage, serving as one of the biomarkers for oxidative stress (Tangvarasittichai, 2015). As shown in Figure 2D, the MDA content in the MR group was significantly higher than that in the MN, MLD, and MHD groups. Combined with the detection results of SOD content, constipation will cause oxidative stress damage to the body, and ZDD has an antioxidant effect.

### 3.4. Effects of different doses of ZDD on the intestinal mucosal microbiota of mice with HPHFD-induced constipation

**Figure 3:**
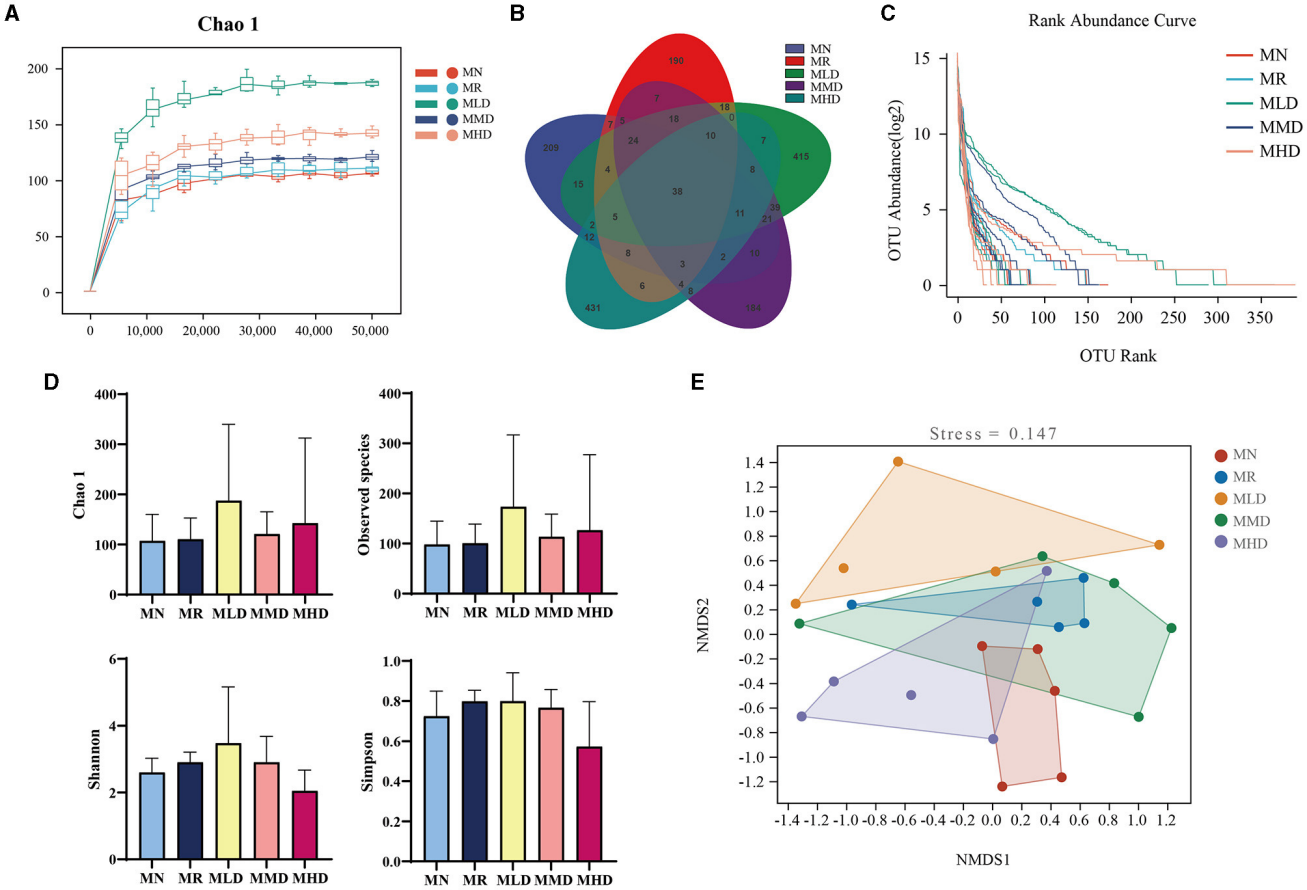
Changes in the ASV numbers and diversity of intestinal mucosal microbiota in mice. **(A)** Chao 1 dilution curve. **(B)** Venn diagram. **(C)** Rank abundance distribution curve. **(D)** Alpha diversity indices. **(E)** NMDS analysis.

The Chao 1 dilution curve (Figure 3A) shows that when the sequencing amount of each sample reaches 2,000, the curve enters a plateau period, and the microbial amount detected in each sample is close to saturation. It shows that the current sequencing depth is enough to reflect the microbial diversity in this sample batch. The total ASV numbers of MN, MR, MLD, MMD, and MHD groups were 209, 290, 415, 184, and 431, respectively. The ASV number of the five groups that generated the intersection was 38 (Figure 3B). It can be seen that MHD group has the largest number of ASVs, which is consistent with the ASV of the MHD group in the Venn diagram above (Figure 3C). Figure 3D shows the Alpha diversity index. Compared with the MN group, the Chao1 indices and observed species had different degrees of increase in the MR, MLD, MMD, and MHD groups. Compared with the MN group, the Shannon and Simpson indices were increased in the MR, MLD, and MMD groups, while the MHD group were decreased, though not significantly, which needs further analysis through the structure of the intestinal microbiota. In NMDS analysis (Figure 3E), there is no intersection between the MN and MR groups, which indicates that constipation induced by HPHFD changes the structure of the intestinal mucosal microbiota. The groups that had the intersection with MN and were closest to the MMD and MHD groups indicated that medium and high doses of ZDD were good for the recovery of the intestinal mucosal microbiota structure.

### 3.5. Effect of different doses of ZDD on the predominant microbiota in the intestinal mucosa of mice with HPHFD-induced constipation

**Figure 4:**
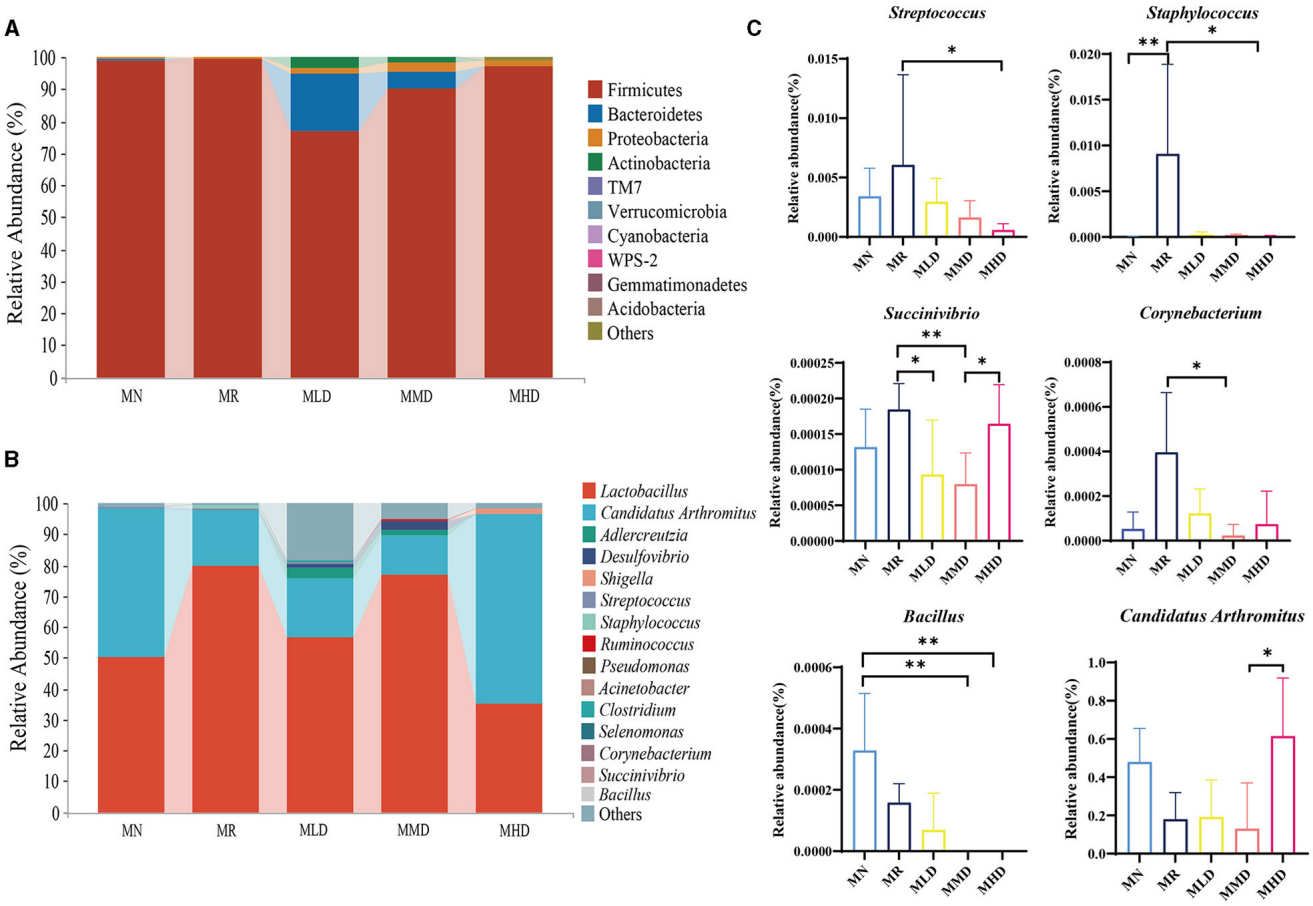
Dominant microbiota of intestinal mucosa in mice. **(A)** Phylum level. **(B)** Genus level. **(C)** Differential bacteria in genus level. *n* = 5, *p < 0.05, **p < 0.01.

At the phylum level (Figure 4A), Firmicutes is the dominant phylum in each group, accounting for 98.57, 99.21, 76.87, 90.32, and 96.91% in the MN, MR, MLD, MMD, and MHD groups, respectively, followed by Bacteroidetes, the proportions were 0.61, 0.32, 18.01, 4.82, and 0.13%, respectively. The third was Proteobacteria, with proportions of 0.57, 0.20, 1.49, 2.99, and 1.56%, respectively.

At the genus level (Figure 4B), the dominant bacterium in each group was *Lactobacillus*, and the proportion in the MN, MR, MLD, MMD, and MHD groups was 50.05, 79.53, 56.61, 76.68, and 35.36%, respectively, followed by *Candidatus Arthromitus*, accounting for 47.96, 17.92, 19.11, 13.05, and 61.44% in the MN, MR, MLD, MMD, and MHD groups, respectively. In addition, *Adlercreutzia* was abundant in the MLD and MHD groups, accounting for 3.32% in MLD group and 1.59% in MMD group.

Then, the above species were statistically analyzed (Figure 4C), and the relative abundance of *Streptococcus* in MR group was significantly higher than that in the MHD group (*p* < 0.05) and that of *Staphylococcus* was significantly higher than that in the MHD group (*p* < 0.05) and MN (*p* < 0.01). The abundance of *Succinivibrio* in the MR groups was significantly higher than that in the MLD (*p* < 0.05) and MHD groups (*p* < 0.01), while in the MHD group, it was significantly higher than that in the MMD group (*p* < 0.05). Compared to the MMD group, the relative abundance of *Corynebacterium* in the MR group and *Candidatus Arthromitus* in the MHD group was significantly increased (*p* < 0.05). *Bacillus* in the MN group was significantly higher than that in the MMD (*p* < 0.01) and MHD groups (*p* < 0.01).

### 3.6. Effects of different doses of ZDD on the intestinal mucosal characteristic microbiota of mice with HPHFD-induced constipation

**Figure 5:**
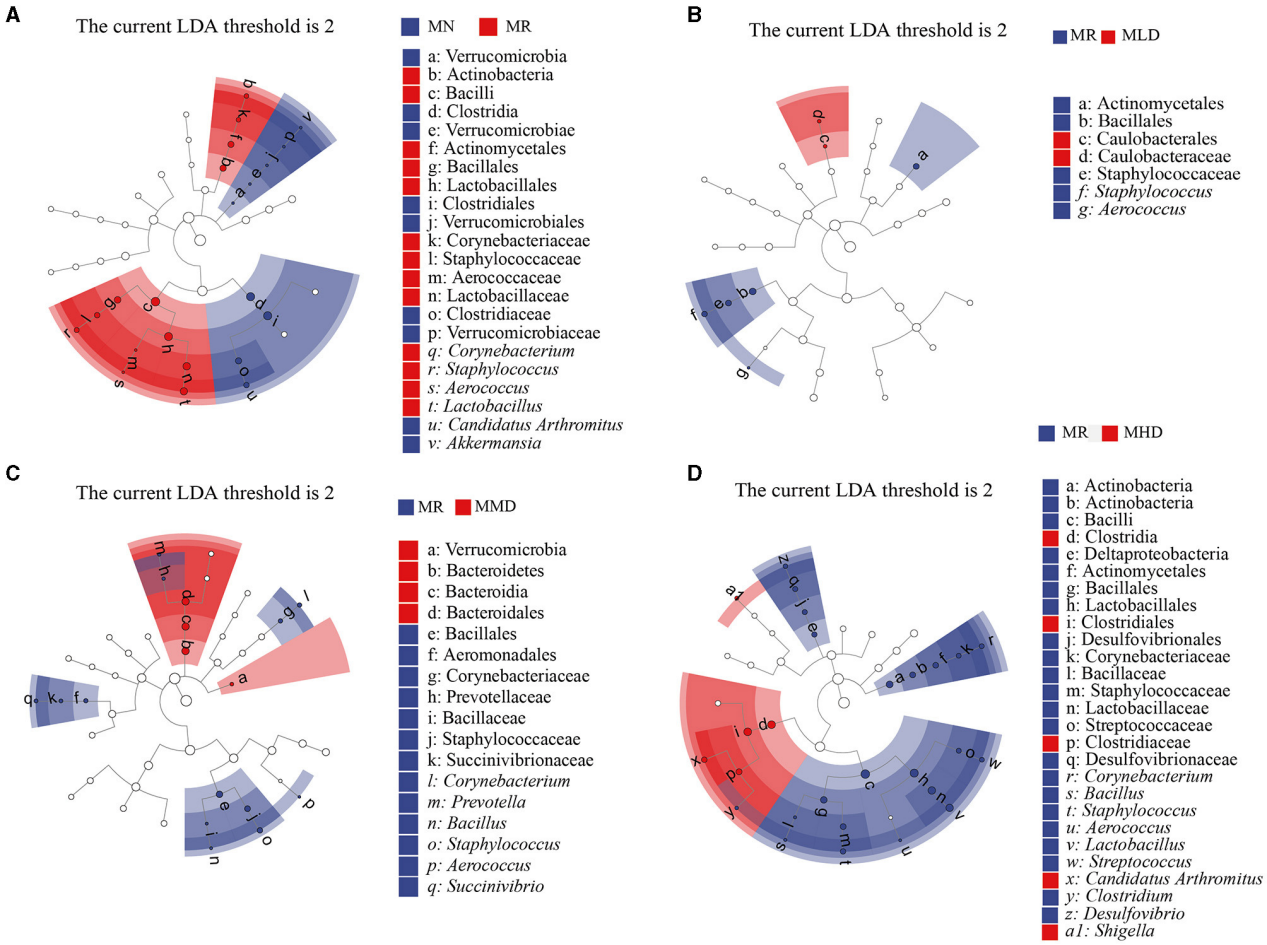
Characteristic bacteria of intestinal mucosa in mice. **(A)** MN and MR. **(B)** MR and MLD. **(C)** MR and MMD. **(D)** MR and MHD.

We selected the LEfSe at a logarithmic LDA threshold of 2.0 to identify microbiota that differed significantly. We mainly analyzed the characteristic bacteria between the MN and MR, MR, and MLD, MR and MMD, MR, and MHD groups. In the LEfSe analysis between the MN and MR groups (Figure 5A), *Lactobacillus, Aerococcus, Staphylococcus*, and *Corynebacterium* were the characteristic bacteria of the MR group, while *Akkermansia* and *Candidatus Arthromitus* belong to the characteristic bacteria of the MN group. In the LEfSe analysis between the MR and MLD groups (Figure 5B), *Aerococcus* and *Staphylococcus* were mainly enriched in the MR group. In the LEfSe analysis between the MR and MMD groups (Figure 5C), *Succinivibrio, Aerococcus, Staphylococcus, Bacillus, Prevotella*, and *Corynebacterium* were characteristic bacteria in the MR group. In the LEfSe analysis between the MR and MHD groups (Figure 5D), the characteristic bacteria of the MR group were *Desulfovibrio, Clostridium, Streptococcus, Lactobacillus, Aerococcus, Staphylococcus, Bacillus*, and *Corynebacterium*, and the characteristic bacteria of the MHD group were *Shigella* and *Candidatus Arthromitus*.

### 3.7. Analysis of different doses of ZDD on the ROC curve

**Figure 6:**
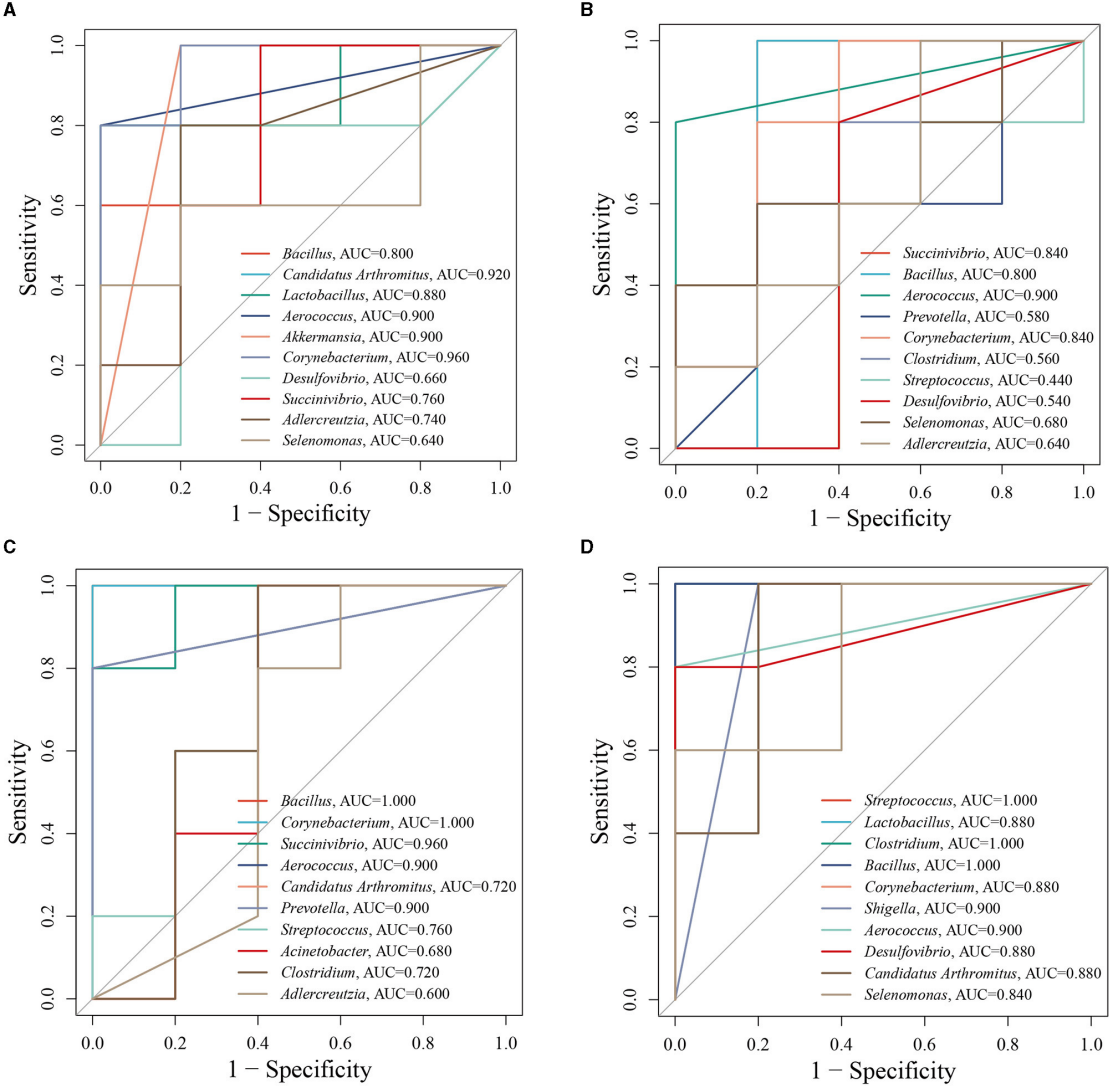
ROC curve analysis. **(A)** MN and MR. **(B)** MR and MLD. **(C)** MR and MMD. **(D)** MR and MHD.

To further analyze the influence of different doses of ZDD on the correlation between intestinal mucosal microbiota and indexes in constipation mice, we established a random forest model to screen the top eight important bacteria in the MR and MLD, MR and MMD, and MR and MHD groups and conducted ROC analysis on these bacteria. We used an AUC of >0.8 as the standard to verify the accuracy of the diagnosis and the joint evaluation of characteristics. Using ROC analysis, different groups can determine whether they have diagnostic efficacy (Li et al., 2023). In the analysis of the MN and MR groups (Figure 6A), the bacteria with an AUC of >0.8 were *Corynebacterium* (AUC = 0.960), *Akkermansia* (AUC = 0.900), *Aerococcus* (AUC = 0.900), *Lactobacillus* (AUC = 0.880), *Candidatus Arthromitus* (AUC = 0.920), and *Bacillus* (AUC = 0.800). For the analysis involving the MLD and MR groups (Figure 6B), the following bacterial species demonstrated prominent AUC values: *Corynebacterium* (AUC = 0.840), *Aerococcus* (AUC = 0.900), *Bacillus* (AUC = 0.800), and *Succinivibrio* (AUC = 0.840). Similarly, in the assessment of the MMD and MR groups (Figure 6C), specific bacterial species stood out with noteworthy AUC scores: *Prevotella* (AUC = 0.900), *Aerococcus* (AUC = 0.900), *Succinivibrio* (AUC = 0.960), *Corynebacterium* (AUC = 1.000), *Bacillus* (AUC = 1.000). Finally, in the evaluation of the MHD and MR groups (Figure 6D) several bacterial species were characterized by significant AUC values: *Desulfovibrio* (AUC = 0.880), *Aerococcus* (AUC = 0.900), *Shigella* (AUC = 0.900), *Corynebacterium* (AUC = 0.880), *Bacillus* (AUC = 1.000), *Clostridium* (AUC = 1.000), *Lactobacillus* (AUC = 0.880), *Streptococcus* (AUC = 1.000), *Candidatus Arthromitus* (AUC = 0.880), and *Selenomonas* (AUC = 0.840). Among them, the AUC of *Corynebacterium, Akkermansia, Aerococcus, Bacillus, Succinivibrio, Desulfovibrio, Shigella, Clostridium, Lactobacillus, Selenomonas*, and *Candidatus Arthromitus* was >0.8, and we believe they are diagnostic bacteria.

### 3.8. Correlation of mice with HPHFD-induced constipation

**Figure 7:**
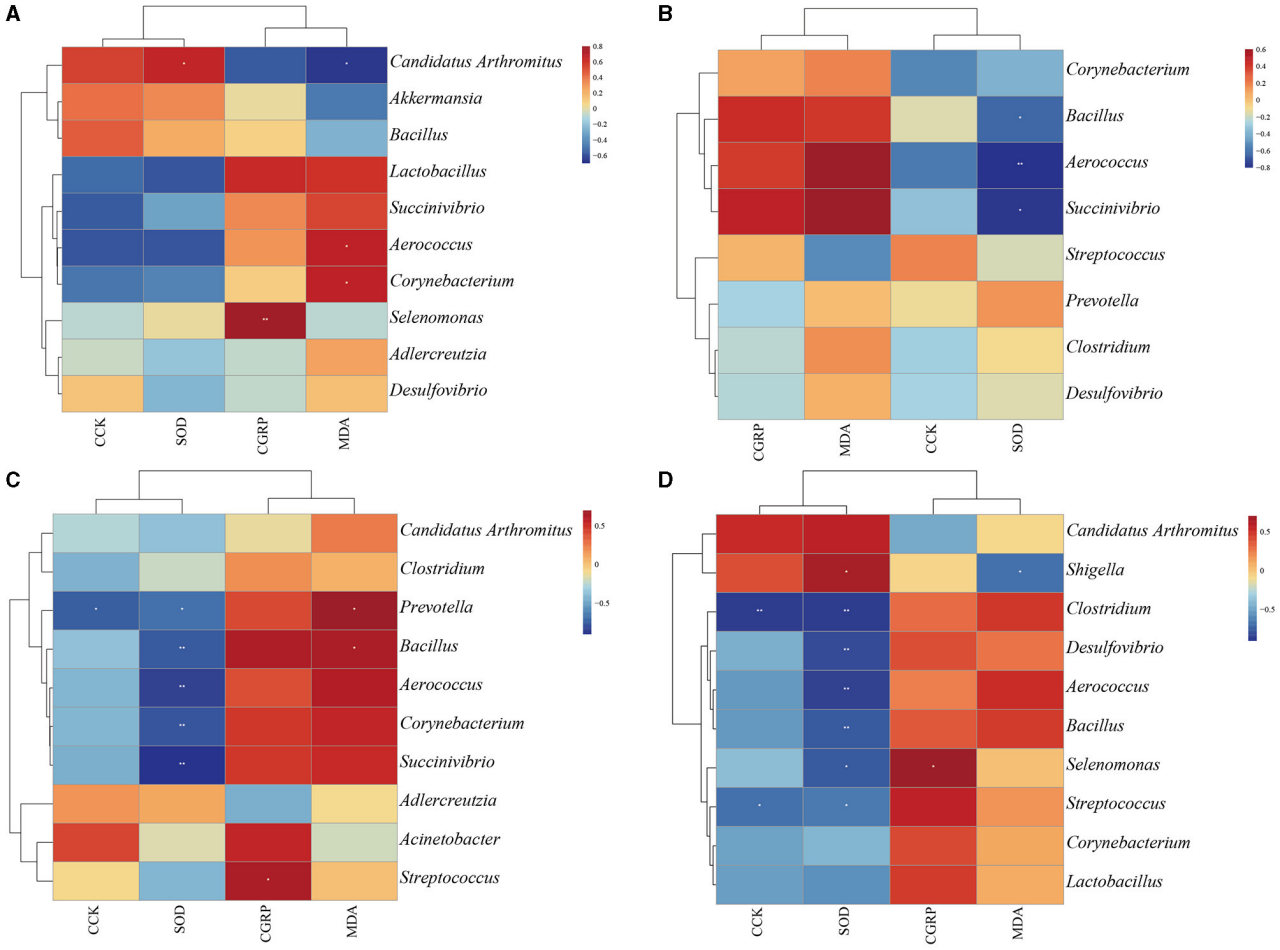
Heat maps of correlation between characteristic bacteria, CCK, CGRP, MDA, and SOD. **(A)** MN and MR. **(B)** MR and MLD. **(C)** MR and MMD. **(D)** MR and MHD. *n* = 5, ^*^P < 0.05, ^**^P < 0.01.

Then, we selected the top eight characteristic genera in the MR and MLD, MR and MMD, and MR and MHD groups by random forest analysis and directly analyzed the correlation with CCK, CGRP, MDA, and SOD. In the analysis of the top eight characteristic bacteria and related indicators of the MN and MR groups (Figure 7A), *Candidatus Arthromitus* showed a positive correlation with SOD (*p* < 0.05) and a significant negative correlation with the MDA (*p* < 0.05). At the same time, the MDA groups was positively correlated with *Corynebacterium* (*p* < 0.05) and *Aerococcus* (*p* < 0.05). Compared with the MR and MLD groups (Figure 7B), SOD had a significantly negative correlation with *Aerococcus* (*p* < 0.05) and *Bacillus* (*p* < 0.01). Compared with the MR and MMD groups (Figure 7C), *Prevotella* showed a significantly negative correlation with CCK (*p* < 0.05). *Succinivibrio* (*p* < 0.05), *Corynebacterium* (*p* < 0.01), *Aerococcus* (*p* < 0.01), *Bacillus* (*p* < 0.01), and *Prevotella* (*p* < 0.01) were negatively correlated with SOD. There was a significant positive correlation between CGRP and *Streptococcus* (*p* < 0.05). The MDA was positively correlated with *Bacillus* (*p* < 0.05) and *Prevotella* (*p* < 0.05). In the analysis of the top eight characteristic bacteria and related indicators of the MR and MHD groups (Figure 7D), CCK was negatively correlated with *Clostridium* (*p* < 0.01), and *Streptococcus* (*p* < 0.05). SOD was positively correlated with *Shigella* (*p* < 0.05) and negatively correlated with *Bacillus* (*p* < 0.01), *Aerococcus* (*p* < 0.01), *Desulfovibrio* (*p* < 0.01), *Clostridium* (*p* < 0.01), and *Streptococcus* (*p* < 0.05). MDA was negatively correlated with *Shigella* (*p* < 0.05).

### 3.9. Analysis of different doses of ZDD on the functions of intestinal mucosal microbiota of mice with HPHFD-induced constipation

**Figure 8:**
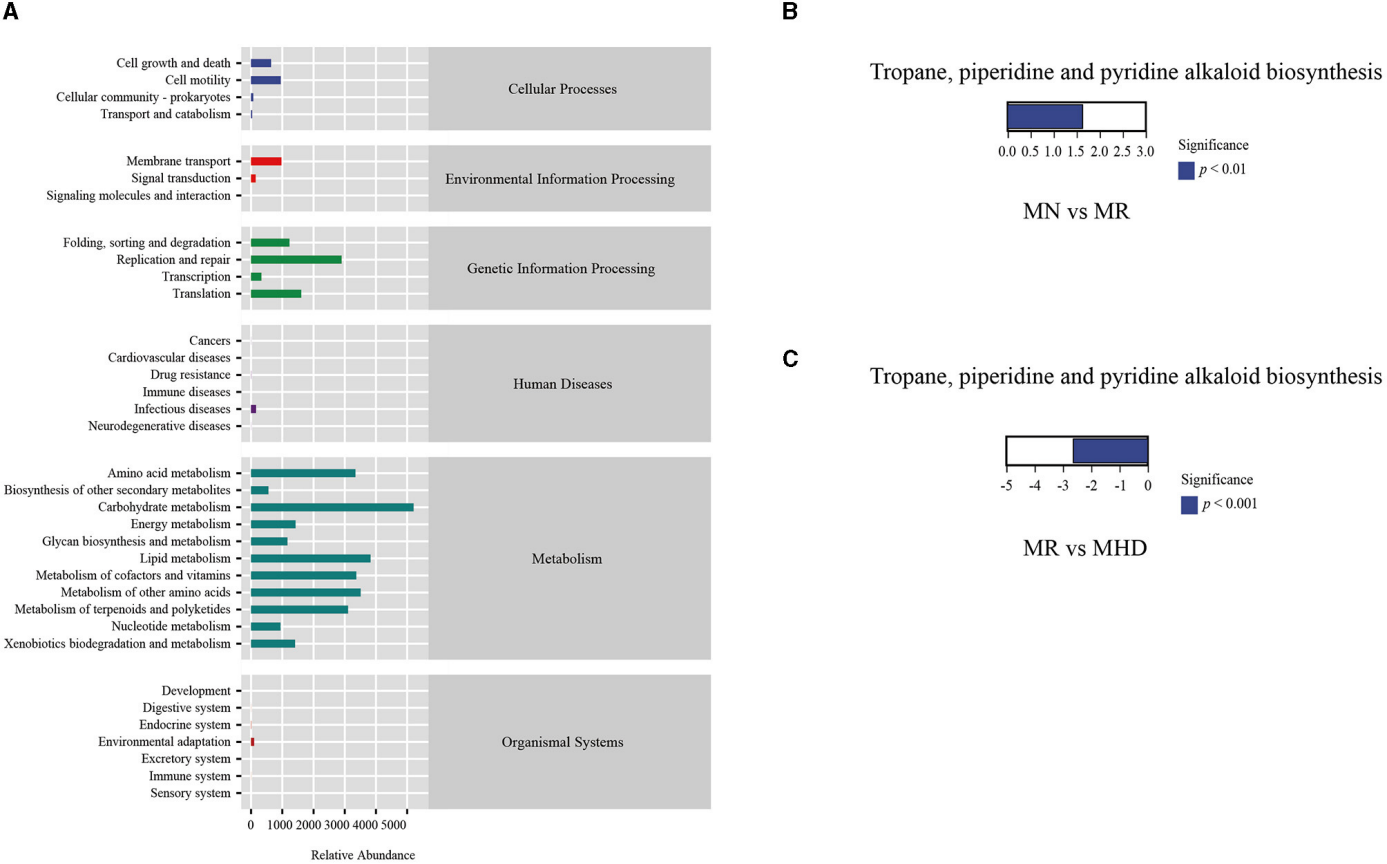
Functional prediction analysis. **(A)** KEGG metabolic pathway. **(B)** The differential metabolic pathway between MN and MR. **(C)** The differential metabolic pathway between MR and MHD (up-regulated group vs. control group, the positive horizontal axis in the figure represents up-regulated group compared with the control group, while the negative value is down-regulated).

The functions of the intestinal mucosal microbiota were generally divided into six categories (Figure 8A): organismal systems, metabolism, human diseases, genetic information processing, environmental information processing, and cellular processes. The second level includes 35 sub-functional classes. The abundance of Cell motility, Cell growth, and Cell death was high in the Cellular processes' metabolic pathway. In the realm of Environmental Information Processing, Membrane transport is highly abundant. Translation, Replication and repair, Folding, sorting and degradation exhibit a highly abundant presence in Genetic Information Processing. In addition, Metabolisms of terpenoids and polyketides, Metabolism of other amino acids, Metabolisms of co-factors and vitamins, Lipid metabolism, Carbohydrate metabolism, and Amino acid metabolism are relatively abundant. Further analysis (Figures 8B, C) shows significant upregulation in MN group relative to MR group (*p* < 0.05) and significant downregulation in MR group relative to MHD group (*p* < 0.05) within the abundance of pathways associated with tropone, piperidine, and pyrroline alkaloid biosynthesis.

## 4. Discussion

Clinically, it has been proven that ZDD can relieve constipation caused by HFHPD. Its mechanism is related to the recovery of intestinal transport function and the transformation of the spleen and stomach (Hong and Hong, 2014). This will inevitably interact with the flora in the intestine and participate in the therapeutic mechanism of drugs.

The body weight of the mice fed HFHPD combined with LOP decreased compared to that of MN group. Combined with the fecal characteristics and weight changes of the mice, we considered that HFHPD would lead to constipation, dyspepsia, and less nutrition absorption in mice. After the administration of ZDD, the body weight of mice increased compared with MR group, but it was not significant. Moreover, the body weight of the mice in the MHD group was close to that of mice in the MN group. We speculate that ZDD can relieve dyspepsia caused by HFHPD modeling, with high-dose ZDD having the most prominent effect.

We further analyzed the levels of gastrointestinal hormones and oxidative stress. The results of CCK and CGRP indicated that the intestinal peristalsis of MR group was lower than that of the mice in the MN, MLD, MMD, and MHD groups. The results of the analysis of MDA and SOD showed the presence of oxidative stress damage in constipation mice induced by HFHPD. After the ZDD intervention, the oxidative stress injury in mice was mitigated to a level akin to that observed in the MN group. These findings suggest that ZDD may exert antioxidant properties and yield beneficial effects in counteracting oxidative stress injuries.

Intestinal mucosal symbiotic microbiota is a part of the intestinal mucosal barrier, which means that intestinal mucosal microbiota is more closely related to intestinal epithelial cells and intestinal mucosal function than fecal microbiota (Codling et al., 2010). Therefore, we analyzed the intestinal mucosal microbiota of mice. From the Chao 1 dilution curve analysis, we believe that the experimental samples meet the requirements of microbial diversity analysis. ASV analysis showed that the HFHPD constipation model and the ZDD intervention could alter the intestinal mucosal microbiota in mice. In the grouping of NMDS, the HFHPD model mice and the ZDD intervention changed the intestinal mucosal microbiota structure, and the distance between MMD and MHD groups was closer to MN group. It indicated that medium and high doses of ZDD can restore the intestinal mucosal microbiota's structure. Therefore, by comparing the relative abundance between groups, we can further understand the influence of ZDD on the intestinal microbial environment.

The relative abundance of *Candidatus Arthromitus* in MHD group was higher than that in MMD, and *Candidatus Arthromitus* has a protective effect on the intestine (Ma et al., 2019; Hou et al., 2022). The relative abundance of *Staphylococcus* and *Streptococcus* in MR was significantly higher than that in MHD group. *Staphylococcus* infection can damage the intestinal barrier and the structure of immune organs (Liu G. et al., 2020). *Streptococcus* can cause inflammatory reactions (Batista and Ferreira, 2015; Hashizume-Takizawa et al., 2019). It indicated that HFHPD-induced constipation can increase the relative abundance of pathogenic bacteria. The relative abundance of *Succinivibrio* in MR group was significantly higher than that in the MLD and MMD groups, while that in the MHD group was significantly higher than that in the MMD group. This may be due to the dose-effect relationship of negative feedback formed by high doses of traditional Chinese medicine, which leads to an increase in *Succinivibrio*. It may also be due to the influence of the overall environment. In a high-dose environment, the reproduction of *Succinivibrio* can be promoted by stimulating a certain bacterium. *Succinivibrio* can cause inflammatory reactions (Marquez-Ortiz et al., 2023). *Corynebacterium* was enriched in MR. *Corynebacterium* can not only damage intestinal microbial barrier function but also cause inflammatory reactions (Yang et al., 2022). In addition, *Bacillus* can inhibit the proliferation of harmful bacteria by consuming oxygen in the intestinal tract or secreting antibacterial substances, thereby regulating intestinal microbiota disorders (Père and Etienne, 2007; Aly et al., 2008). However, after ZDD intervention, the relative abundance of *Bacillus* was lower than that in MN group, which may be related to the complex chemical components in the prescription.

We further compared the other groups with MR group by LEfSe analysis and found that MR group was rich in pathogenic bacteria, such as *Aerococcus, Corynebacterium, Desulfovibrio, Clostridium*, and *Prevotella*. Among them, *Aerococcus* is a common harmful bacterium that can lead to an imbalance in the intestinal microbiota (Zheng et al., 2022). *Desulfovibrio* can produce H_2_S that is harmful to the human body and has a pro-inflammatory effect (Figliuolo et al., 2017). *Clostridium* can increase the risk of gastrointestinal inflammatory reactions (Wang et al., 2022). *Prevotella* colonization leads to metabolic changes in the microbial population and reduces the production of interleukin-18, thus aggravating intestinal inflammation and potential systemic autoimmune disease (Iljazovic et al., 2021). Therefore, constipation induced by HFHPD can increase the relative abundance of pathogenic bacteria in the intestinal mucosa, and the administration of ZDD can reduce the relative abundance of these pathogenic bacteria and relieve constipation by reducing their proliferation.

As we all know, traditional Chinese medicine prescriptions contain many chemical components, as does ZDD. Experimental studies show that hesperidin and baicalin are anti-inflammatory and anti-bacterial active components in ZDD, which significantly regulate gastrointestinal movement (Tan et al., 2006; Liu et al., 2023). Hesperidin can selectively remove harmful intestinal microbiota and regulate intestinal microbiota disorders through bacteriostasis, thus affecting intestinal peristalsis. The antibacterial mechanism of hesperidin may be related to its chemical structure, which can coagulate or denature proteins (Guardia et al., 2001). It was found that hesperidin can reduce the relative abundance of harmful bacteria such as *Staphylococcus* and *Desulfovibrio*, which is similar to our experimental results (Mas-Capdevila et al., 2020). Baicalin can regulate the structure of the intestinal microbiota, inhibit gram-negative and positive bacteria, and reduce endotoxin entering the blood and the secretion of inflammatory factors, thus alleviating metabolic inflammation, protecting intestinal epithelial cells from damage, improving intestinal mucosal structure, and promoting intestinal peristalsis (Zhang X. Y. et al., 2021). Our experimental results show that the relative abundance of Gram-positive bacteria such as *Staphylococcus, Streptococcus* and Gram-negative bacteria such as *Succinivibrio* decreased to different degrees after different doses of ZDD intervention. Therefore, we speculate that hesperidin and baicalin play a key role in inhibiting harmful bacteria in ZDD.

Among the characteristic bacteria enriched in MR group, it was found that *Corynebacterium, Aerococcus, Bacillus*, and *Prevotella* were positively correlated with MDA. *Streptococcus* was positively correlated with CGRP. *Succinivibrio, Corynebacterium, Aerococcus, Bacillus, Prevotella, Clostridium*, and *Streptococcus* were negatively correlated with SOD. *Prevotella, Clostridium*, and *Streptococcus* are negatively correlated with CCK. These correlations may be one of the mechanisms of constipation induced by HFHPD. *Candidatus Arthromitus* has a negative correlation with MDA and a positive correlation with SOD, which is similar to the research results of Chen et al. (2023). We speculate that *Candidatus Arthromitus* may relieve constipation by regulating the level of oxidative stress to restore intestinal mucosal dysfunction caused by intestinal mucosal cell damage. In addition, ZDD may reduce the secretion of CGRP to decrease the inhibition of gastrointestinal movement and improve intestinal peristalsis. ZDD can also stimulate intestinal peristalsis and relieve constipation by increasing the secretion of CCK. The mechanism between them needs further study. We also performed functional prediction on intestinal mucosal bacteria and found that, compared with MR group, the abundance of tropone, piperidine, and pyrroline alkaloid biosynthesis pathways was significantly up-regulated in MN and MHD groups. We speculate that tropane, piperidine, and pyridine alkaloid biosynthesis is one of the directions of ZDD to relieve constipation caused by HFHPD.

## 5. Conclusion

In summary, constipation caused by HFHPD could increase the relative abundance of pathogenic bacteria in the intestinal mucosal microbiota. After the intervention of ZDD, constipation was relieved, and the relative abundance of pathogenic bacteria was reduced. In particular, the high dose of ZDD can not only reduce the oxidative stress damage and increase the relative abundance of beneficial bacteria such as *Candidatus Arthromitus* but also increase the abundance of tropane, piperidine, and pyridine alkaloid biosynthesis, which is of great significance for the treatment of constipation.

## Data availability statement

The datasets presented in this study can be found in online repositories. The names of the repository/repositories and accession number(s) can be found below: https://www.ncbi.nlm.nih.gov/, PRJNA 963077.

## Ethics statement

The animal study was approved by Animal Care and Use Committee of the Hunan University of Chinese Medicine. The study was conducted in accordance with the local legislation and institutional requirements.

## Author contributions

XP: data analysis and writing the original draft. XY and JL: performing animal experiments. ND and ZT: review and editing. YC and ZT: project administration, review, and funding acquisition. All authors contributed to the article and approved the submitted version.
